# Apolipoprotein E and kidney function in older adults 

**DOI:** 10.5414/CN107427

**Published:** 2012-02-20

**Authors:** Rebecca Kurnik Seshasai, Ronit Katz, Ian H. de Boer, David Siscovick, Michael G. Shlipak, Dena E. Rifkin, Mark J. Sarnak

**Affiliations:** 1 Division of Nephrology, Department of Medicine, Tufts Medical Center, Boston, MA,; 2 Collaborative Health Studies Coordinating Center,; 3 Division of Nephrology, Department of Medicine,; 4 Departments of Medicine and Epidemiology, University of Washington, Seattle, WA,; 5 General Internal Medicine Section, San Francisco VA Medical Center and Departments of Medicine, Epidemiology and Biostatistics, University of California, San Francisco, and; 6 Division of Nephrology, Department of Medicine, University of California, San Diego, CA, USA

**Keywords:** apolipoprotein E, chronic kidney disease, kidney function, elderly

## Abstract

Background: Previous studies suggest that the ε4 and ε2 alleles of apolipoprotein E (*APOE*) may be associated with decreased and increased risks of CKD, respectively, but there are limited data in older adults. We evaluated the associations of apolipoprotein E alleles with kidney function among older adults in the cardiovascular health study (CHS). Methods: Caucasian participants had APOE allelic analysis and serum creatinine and cystatin C measured at baseline (n = 3,844 for cross sectional analysis) and in follow up (n = 3,226 for longitudinal analysis). *APOE* variation was evaluated as an additive model with number of ε2, ε3 and ε4 alleles. GFR was estimated using the CKD epidemiology equation (eGFRcreat) and the cystatin C demographic equation (eGFRcys). The primary outcome was CKD defined by eGFR < 60 ml/min/1.73 m^2^. The secondary outcome was rapid progression defined by annual loss of eGFR > 3 ml/min/1.73 m^2^. Results: Mean eGFRcreat was 72 ml/min/1.73 m^2^ (25% CKD). Compared with the ε3 allele, the *APOE* ε4 allele was associated with reduced risk of CKD by eGFRcreat: unadjusted odds ratio (OR) and 95% confidence interval (CI) 0.79 (0.67 – 0.93) per allele, fully adjusted OR (95% CI) 0.80 (0.68 – 0.96) per allele. Results were consistent using eGFRcys. There was no association of the ε2 allele with CKD or between the apolipoprotein E gene with rapid progression. Conclusions: The apolipoprotein ε4 allele was associated with lower odds of CKD in elderly Caucasian individuals. Future research should confirm these findings in other races and explore mechanisms to explain these results.

## Introduction 

Chronic kidney disease (CKD) is a public health problem which predominately affects older adults. More than 30% of individuals older than 70 years have CKD, defined by an estimated glomerular filtration rate (eGFR) lower than 60 ml/min/1.73 m^2^ [1]. Many elements contribute to the development and progression of CKD including genetic variation, diabetes, hypertension, and dyslipidemia. Specifically, elevated triglycerides and low high density lipoprotein (HDL) cholesterol have been associated with CKD in several studies [2]. Relatively little is known however about the risk factors for CKD in the elderly. 

Apolipoprotein E (*APOE*), a constituent of plasma lipoproteins, has 3 alleles (ε2, ε3, ε4) where ε3 is seen most frequently in the population [3]. The ε4 allele is an established risk factor for Alzheimer’s disease [3] and is also associated with coronary heart disease [4]. The latter is thought perhaps due to an effect of the ε4 allele in increasing low density lipoprotein cholesterol (LDL) cholesterol. In contrast the ε4 allele may be associated with a lower risk of diabetic nephropathy [5]. A potential mechanism to explain the associations with CKD include modulation of lipoprotein metabolism as increased ε4 is associated with higher levels of HDL cholesterol and lower levels of triglycerides [3], a lipid profile which in particular may decrease risk of CKD [2]. The ε2 allele in turn is associated with Type III hyperlipoproteinemia and increased levels of triglycerides [6], which may promote progression of kidney disease. Independent of their lipid related effects; *APOE* alleles may also have allelic varying actions on vascular smooth muscle and mesangial cell proliferation [7]. The relationship between allelic frequency and disease also appears to vary by race and ethnicity [8]. 

There are few data on the relationship of apolipoprotein E with CKD. Some [9, 10, 11], but not all [12, 13], small cross sectional studies suggest that ε2 is associated with higher and ε4 with lower risk of CKD compared with the ε3 allele. In studies among persons with diabetes, those with the ε2 allele were more likely to have macroalbuminuria [9] and worse kidney function, whereas those with the ε4 allele had a lower prevalence of diabetic nephropathy and higher levels of glomerular filtration [5, 10]. Similarly, among patients with end stage renal disease (ESRD), there was higher allelic frequency of ε2 [11] and lower frequency of ε4 compared with controls [11]. A prospective study from the atherosclerosis in risk in communities study of middle aged adults demonstrated that the *APOE* ε4 allele was associated with lower risk of kidney disease progression [14]. These results were not mediated by diabetes, hypertension or dyslipidemia. We are not aware of large studies that have evaluated APOE subtypes in older adults, the population who are at highest risk for CKD [1]. 

We evaluated the association of apolipoprotein E allelic frequency with prevalent CKD and rate of progression of kidney disease in older adults in the Cardiovascular Health Study [15]. We hypothesized that the ε2 and ε4 alleles of *APOE* would increase and decrease the risk of CKD and its progression, respectively. To evaluate the consistency of the results we also evaluated cystatin C as the measurement of kidney function, as cystatin C appears to be less dependent on muscle mass and thus may be a more accurate measure of kidney function in the elderly [16]. 

## Subjects and methods 

### Study population 

The Cardiovascular Health Study (CHS) is a community-based prospective cohort study of cardiovascular disease of persons 65 years or older at the beginning of the study in 1989. It was designed to evaluate risk factors for cardiovascular disease and stroke [15]. Briefly, 5,201 men and women 65 years or older who were ambulatory and living in the community were randomly selected and enrolled from Medicare eligibility lists in Forsyth County, NC; Sacramento County, CA; Washington County, MD; and the city of Pittsburgh, PA, USA. An additional 687 African American participants were recruited and enrolled in 1992 – 1993. Subjects were excluded if they were institutionalized, home-bound, receiving hospice, radiation, or chemotherapy for cancer, unable to give informed consent, or were planning to move out of the area within 3 years. Full details of the study design are previously described [15]. All participants provided written informed consent, and all CHS sites approved the study. 

*APOE* analysis was performed in 5,494 individuals. Our study population was limited to Caucasian participants given the genetic nature of the study and the limited statistical power to adequately evaluate the relationship in African Americans [8]. We also restricted to individuals with both creatinine and cystatin C measured at baseline. This resulted in 3,844 participants for the cross sectional analysis. Participants with at least two measurements of cystatin C and serum creatinine (n = 3,226) were included in the longitudinal analysis. 

### Exposure 

The three allelic forms of the *APOE* gene were genotyped in the core molecular genetics facility at the University of Vermont College of Medicine by the method of Hixson and Vermier as previously described [17, 18]. 

### Outcome 

Measurement of cystatin C and creatinine: Frozen sera stored at –70 °C from the visits at baseline (1989 – 1990), Year 3 (1992 – 1993) and Year 7 (1996 – 1997) were available for measurement of cystatin C. Cystatin C was measured using a particle-enhanced immunonephelometric assay (N Latex cystatin C, Dade Behring, now Siemens Healthcare Diagnostics Inc., Deerfield, IL, USA) with a nephelometer (BNII, Siemens Healthcare Diagnostics Inc.). For cystatin C, intra-assay coefficients of variation (CVs) range from 2.0 to 2.8% and inter-assay CVs range from 2.3 to 3.1%. Creatinine was measured in batched samples using a colorimetric method (Ektachem 700, Eastman Kodak, Rochester, NY, USA). The mean CV for monthly controls was 1.94 (range 1.16 – 3.60%). 

Primary outcome: The primary outcome was based on the cross sectional analysis as APOE (a genetic risk factor) will have influenced the risk of CKD over the individuals’ lifetime (~ 72 years in this study). This is in contrast to the longitudinal analyses where mean length of follow up was 6.8 years and the gene had a much shorter time to have an effect. GFRcreat was estimated using the CKD Epi formula calculated as follows: GFR = 141 × min(Scr/,1)^α^ × max(Scr/,1)^–1.209^ × 0.993^Age^ × 1.018 (if female) × 1.159 (if black), where Scr is serum creatinine (mg/dl), is 0.7 for females and 0.9 for males, is –0.329 for females and –0.411 for males, min indicates the minimum of Scr/κ or 1, and max indicates the maximum of Scr/ or 1 [19]. eGFRcys was estimated using the following equation. eGFR_cystatin_ c = 127.7 × CysC^1.17^ × age^0.13^ × (0.91 if female) × (1.06 if black) [20]. CKD was defined by eGFR < 60 ml/min/1.73 m^2^ using either the CKD Epi equation [19] or the cystatin C demographic equation [20]. 

Secondary outcome: Our secondary outcome was based on longitudinal analyses. Rates of change were calculated using the two or three available cystatin C and creatinine measurements. Annualized change in eGFR was calculated using a least-squares regression slope. Rapid progression of kidney disease was defined by an annual loss of > 3 ml/min/1.73 m^2^. This magnitude of change is ~ 3 times the expected rate previously described in studies of normal aging [21] and represents the highest quartile of kidney function loss in CHS. Furthermore, it has been used in prior studies of kidney function decline [22], and is an outcome associated with adverse consequences [22]. 

### Covariates 

We chose covariates that may improve precision of the genetic risk estimates. The following covariates were examined: demographic variables (age, gender) and vascular risk factors including body mass index, hypertension (defined by history and use of antihypertensive agents, or an average of three blood pressure measurements greater than 140/90 mmHg), diabetes (defined by use of insulin or an oral hypoglycemic agent, or a fasting blood sugar > 126 mg/dl), smoking (never, former, current), total cholesterol, low density lipoprotein cholesterol, high density lipoprotein cholesterol and triglycerides. 

### Statistical analysis 

Baseline characteristics were compared across apolipoprotein genotypes, ε2/ε2, ε2/ε3, ε2/ε4, ε3/ε3, ε3/ε4, or ε4/ε4. The effects of *APOE* variation were examined as an additive model with number of ε2, ε3 and ε4 alleles modeled separately [14]. Only ε2 and ε4 were entered into the model since ε3 is dependent on the other two and is considered the reference. 

In cross sectional analysis, the associations of ε2, ε3, and ε4 alleles with CKD were evaluated using logistic regression models that were unadjusted, adjusted for age and gender, and fully adjusted. The associations of apolipoprotein E alleles with eGFR on a continuous scale were evaluated using linear regression models that were unadjusted, adjusted for age and gender, and fully adjusted. 

In longitudinal analyses, the associations of ε2, ε3, and ε4 alleles with rapid progression of kidney disease were evaluated using logistic regression models that were unadjusted, age and gender adjusted and fully adjusted. S-Plus (release 6.1, Insightful Inc, Seattle, WA, USA) and SPSS statistical software (release 15.0, SPSS Inc, Chicago, IL, USA) were used for the analyses. All statistical tests were two sided, and p value < 0.05 was considered statistically significant. 

## Results 

### Baseline characteristics 

The mean age of participants was 72 years, 40% were men, 14% had diabetes and 56% had hypertension. Allele frequencies were 16% for ε2, 95.6% for ε3, and 25% for ε4. The predominant genotypes were ε3/ε3 (61.7%), ε3/ε4 (21.1%), and ε2/ε3 (12.8%). The ε4/ε4 group had higher total cholesterol and low density lipoprotein levels. 6% of participants were on lipid lowering meds and 2% were on statins. Mean baseline eGFRcreat was 72 ml/min/1.73 m^2^ and mean eGFRcys was 70 ml/min/1.73 m^2^. 25 – 27% had prevalent CKD (Table 1). 

### Cross sectional analysis 

The ε4 allele was associated with a lower risk of CKD in unadjusted and adjusted analyses using both eGFRcreat and eGFRcys (Table 2). There was no association of the ε2 allele with CKD in unadjusted or adjusted analysis. 

When eGFRcreat was considered on a continuous scale the ε4 allele was associated with higher eGFR in unadjusted and fully adjusted analyses (Table 3). When eGFRcys was considered on a continuous scale, there was a significant association in unadjusted analysis; however, these relationships were not significant in adjusted analysis. There was no association of ε2 with eGFR in unadjusted or adjusted analysis. 

### Longitudinal analysis 

12% and 22% had rapid kidney decline by eGFRcreat and eGFRcys, respectively, over a mean of 6.8 years. Individuals who were excluded from the longitudinal analysis were older, had an increased prevalence of diabetes and hypertension, and lower levels of eGFR. Patients with 2 vs. 3 measures of kidney function however had very similar percent of each of the *APOE* alleles (data not shown). There was no association between either the ε2 or ε4 allele with rapid kidney decline using either eGFRcreat or eGFRcys as the measure of kidney function (Table 4). 

## Discussion 

In this study we demonstrate that the apolipoprotein ε4 allele is associated with lower odds of CKD in older adults. Although there were some small differences based on how kidney function was estimated or whether it was evaluated on a continuous or a dichotomous scale, the results were for the most part consistent. We however found no significant relationship between the ε2 allele and the presence of CKD or between the apolipoprotein E gene and progression of kidney disease. 

To our knowledge, this is the first study evaluating the relationship of apolipoprotein E with CKD in the elderly, as well as the one of the largest cross sectional studies evaluating the relationship of the apolipoprotein E gene with level of kidney function. Consideration of genetic studies of CKD in the elderly is particularly important given the high prevalence of CKD in the elderly, and the fact that genetic associations may be qualitatively different in older versus younger populations because of survival bias. This may be particularly important given that APOE isoforms themselves may be associated with increased risk of dementia and CAD [3, 4] although we are not aware of studies relating APOE isoforms to early mortality. 

For the primary cross sectional outcome, APOE ε4 was associated with lower odds of CKD as well as higher eGFRcreat. These results are consistent with a cross sectional analysis of 5,583 participants in the National Health and Nutrition Examination Survey (NHANES III) where the ε4 allele was negatively associated with low estimated GFR (< 75 ml/min/1.73 m^2^) in non Hispanic Whites [23]. There are several mechanisms through which APOE variation may be associated with lower risk of CKD. These include lipid related and lipid unrelated mechanisms. With regard to the former, APOE is a major protein component of plasma lipoproteins and plays a key role in lipoprotein clearance [3]. Higher levels of apolipoprotein ε4 have been associated with lower triglycerides and higher HDL cholesterol [3]. In turn these abnormalities have been associated with lower risk of progression of CKD [2]. One study evaluating APOE isoforms, plasma lipid levels and remnant lipoproteins demonstrated their differential role in progression of diabetic nephropathy [5]. We did not see much attenuation in multivariable analyses suggesting that at least in our study lipid levels did not modulate the relationship between *Apo E* and CKD. APOE also may have lipid independent affects. APOE is expressed in the kidney, in particular in mesangial cells, where its isoforms may differently regulate growth and survival of mesangial cells and smooth muscle cells. Mesangial cell proliferation and mesangial matrix accumulation have been associated with various forms of kidney disease [7] and in animal models a deficiency of APOE may lead to glomerulosclerosis [7]. 

We noted some discrepancies in the relationships depending on whether eGFRcreat or eGFRcys was used to estimate kidney function. The exact reason for this is unknown but it is important to recognize that each prediction equation identifies a different group with CKD [24]. It is also well recognized that both cystatin C and creatinine have non GFR determinants such as adiposity in the case of cystatin C and muscle mass in the case of creatinine [25], and therefore adjustment for covariates may have a differential effect. 

We were not able to demonstrate that the APOE gene was associated with rapid progression in longitudinal analyses. We suspect that the difference between the cross sectional and longitudinal analyses reflects the difference in duration of follow-up; the longitudinal analyses reflect a relatively short period of follow-up for a genetic condition, whereas the cross sectional analyses in this study reflected longer exposure. Interestingly in the ARIC Study, Hsu et al. [14] did not do cross-sectional analyses but were able to demonstrate differences in progression of kidney disease by APOE genotype in longitudinal analysis. Other potential differences between the two studies include size of the study (n = 14,520 in ARIC), the ARIC cohort being of younger age, different endpoints to define progression of kidney disease, and perhaps most importantly length of follow-up (median of 14 years in ARIC). 

There was no relationship between APOE ε2 and either presence of CKD or progression of kidney disease in our study. This is consistent with a study by Feussner et al. [12], where APOE isoforms were assayed in 560 hemodialysis patients and controls, and no difference in the APOE alleles or APOE phenotypes were noted. Similarly, in 146 patients with insulin dependent diabetes mellitus there were no differences in allele frequencies in those with macroalbuminuria, microalbuminuria and normoalbuminuria [13]. We acknowledge however that the inability to appreciate any significant relationships of the *APOE* ε2 allele may be due to lack of statistical power given the low prevalence of this allele. 

The strengths of this study include the large sample of older adults, detailed ascertainment of risk factors and outcomes in CHS, use of cystatin C as an alternate measure to ascertain level of kidney function, and consideration of cross sectional and longitudinal analysis. There are also several limitations. GFR was not measured directly in CHS and although we utilized the best currently available estimates of GFR, these estimates have not been validated in an elderly cohort. In addition, these results can only be generalized to a Caucasian population. Because CKD in later life reflects a myriad of causes the importance of the APOE allele may be attenuated. We were not able to adjust for level of proteinuria which is an important risk factor for progression of kidney disease. Finally, given the older age of the population, the cross sectional design may be limited by survivor bias, while the longitudinal analysis may have been biased by including a healthier group of individuals than those included in the cross sectional analysis. 

In conclusion, the APOE ε4 allele was associated with lower odds of CKD in older Caucasian adults. Additional study however is needed to confirm this relationship in more diverse populations, and to understand the mechanism through which the APOE ε4 allele leads to lower risk of CKD. 

## Acknowledgments 

The research reported in this article was supported by grants R01-AG-027002, K24DK078204 and contract numbers N01-HC-85239, N01-HC-85079 through N01-HC-85086, N01-HC-35129, N01 HC-15103, N01 HC-55222, N01-HC-75150, N01-HC-45133, grant number HL080295 from the National Heart, Lung, and Blood Institute and grant number AG-023269 from the National Institute on Aging, with additional contribution from the National Institute of Neurological Disorders and Stroke. Additional support was provided through AG-15928, AG-20098, and AG-027058 from the National Institute on Aging, HL-075366 from the National Heart, Lung and Blood Institute, and the University of Pittsburgh Claude D. Pepper Older Americans Independence Center P30-AG-024827. A full list of principal CHS investigators and institutions can be found at http://www.chs-nhlbi.org/pi.htm. Dr. Katz had full access to all of the data in the study and takes responsibility for the integrity of the data and the accuracy of the data analysis. An abstract representing this work was presented at the American Society of Nephrology Annual Meeting in Denver, Colorado in 2010. 

**Table 1.** Baseline characteristics of the study cohort.

	Total	ε2/ε2	ε2/ε3	ε2/ε4	ε3/ε3	ε3/ε4	ε4/ε4	p value
Number (%)	3,844	22 (0.6)	491 (12.8)	99 (2.6)	2,371 (61.7)	810 (21.1)	51 (1.3)	
Age, years	72 ± 5	74 ± 6	73 ± 6	73 ± 5	73 ± 5	72 ± 5	72 ± 5	0.069
Female	60	73	62	65	60	60	59	0.743
BMI (kg/m^2^)	26.3 ± 4.5	26.3 ± 3.8	26.6 ± 4.4	26.0 ± 3.9	26.5 ± 4.5	25.9 ± 4.4	25.8 ± 5.0	0.021
Smoking								p = 0.542
Never	1,775 (46)	11 (50)	226 (46)	50 (51)	1,106 (47)	356 (44)	26 (51)	
Former	1,633 (43)	7 (32)	204 (42)	44 (44)	997 (42)	360 (45)	21 (41)	
Current	436 (11)	4 (18)	61 (12)	5 (5)	268 (11)	94 (12)	4 (8)	
Diabetes	14	14	13	11	15	12	4	0.030
Hypertension	56	68	55	57	57	54	59	0.831
Systolic BP (mmHg)	135 ± 21	141 ± 23	136 ± 20	135 ± 18	135 ± 22	135 ± 21	140 ± 22	0.325
Total cholesterol (mg/dl)	213 ± 39	173 ± 38	200 ± 37	202 ± 38	213 ± 39	218 ± 39	232 ± 45	< 0.001
LDL cholesterol (mg/dl)	131 ± 36	83 ± 27	116 ± 32	120 ± 32	132 ± 35	137 ± 35	144 ± 42	< 0.001
HDL Cholesterol (mg/dl)	54 ± 16	54 ± 14	55 ± 16	52 ± 14	54 ± 16	54 ± 16	57 ± 19	0.250
Triglycerides (IQR)	125 (95, 169)	126 (103, 190)	131 (97, 180)	129 (88, 213)	122 (94, 165)	124 (97, 166)	140 (100,199)	0.058
Lipid lowering medications	6	5	5	2	5	7	12	0.063
eGFRcreat (ml/min/1.73 m^2^)	72 ± 17	71 ± 15	72 ± 18	74 ± 16	72 ± 17	73 ± 17	75 ± 17	0.324
eGFRcys (ml/min/1.73 m^2^)	70 ± 17	66 ± 10	69 ± 17	71 ± 17	69 ± 17	70 ± 17	74 ± 18	0.187
CKD eGFR < 60 (ml/min/1.73 m^2^)								
eGFRcreat	25	23	26	22	26	22	18	0.165
eGFRcys	27	27	28	23	28	24	20	0.094

Values expressed as mean ± standard deviation (SD) for continuous data and percentages for dichotomous data.

**Table 2.** Cross-sectional associations of APOE alleles with prevalent CKD.

	APOE allele OR (95% CI)		
	ε3	ε2	ε4
eGFRcreat			
N	2,470	513	861
#CKD	634	132	184
Unadjusted	1.00 (reference)	0.99 (0.81, 1.20)	0.79 (0.67, 0.93)
Age + gender adjusted	1.00 (reference)	0.97 (0.79, 1.18)	0.82 (0.69, 0.98)
Fully adjusted*	1.00 (reference)	1.01 (0.82, 1.25)	0.80 (0.68, 0.96)
eGFRcys			
N	2,470	513	861
#CKD	693	143	200
Unadjusted	1.00 (reference)	0.97 (0.81, 1.17)	0.77 (0.66, 0.91)
Age + gender adjusted	1.00 (reference)	0.92 (0.75, 1.12)	0.81 (0.68, 0.96)
Fully adjusted*	1.00 (reference)	0.88 (0.71, 1.09)	0.83 (0.69, 0.99)

*Fully adjusted model includes age, gender, diabetes, systolic blood pressure, diastolic blood pressure, antihypertensive medications, body mass index and prevalent coronary heart disease, low density lipoprotein, high density lipoproteins, triglycerides, lipid lowering medication use, and smoking status.

**Table 3.** Associations of APOE alleles with baseline eGFR.

	APOE allele β (95% CI)		
	ε3	ε2	ε4
N	2,470	513	861
eGFRcreat			
Unadjusted	0.00 (reference)	0.17 (–1.27, 1.61)	1.48 (0.30, 2.66)
Age + gender adjusted	0.00 (reference)	0.34 (–1.00, 1.67)	0.93 (–0.16, 2.02)
Fully adjusted*	0.00 (reference)	–0.07 (–1.40, 1.27)	1.07 (0.01, 2.15)
eGFRcys			
Unadjusted	0.00 (reference)	–0.31 (–1.73, 1.11)	1.48 (0.32, 2.64)
Age + gender adjusted	0.00 (reference)	0.07 (–1.26, 1.41)	0.93 (–0.16, 2.03)
Fully adjusted*	0.00 (reference)	0.21 (–1.07, 1.48)	0.53 (–0.50, 1.56)

*Fully adjusted model includes age, gender, diabetes, systolic blood pressure, diastolic blood pressure, antihypertensive medications, body mass index and prevalent coronary heart disease, low density lipoprotein, high density lipoproteins, triglycerides, lipid lowering medication use, and smoking status.

**Table 4:** Associations of APOE alleles with rapid progression.

	APOE allele OR (95% CI)		
	ε3	ε2	ε4
eGFRcreat > 3 ml/min/1.73 m^2^/year			
N	2,179	437	710
#with rapid decline	248	49	79
Unadjusted	1.00 (reference)	0.91 (0.68, 1.22)	0.89 (0.69, 1.13)
Age + gender adjusted	1.00 (reference)	0.88 (0.66, 1.19)	0.91 (0.71, 1.16)
Fully adjusted*	1.00 (reference)	0.87 (0.64, 1.17)	0.95 (0.74, 1.22)
eGFRcys > 3ml/min/1.73 m^2^/year			
N	2179	437	710
#with rapid decline	450	99	158
Unadjusted	1.00 (reference)	1.08 (0.87, 1.34)	1.13 (0.95, 1.36)
Age + gender adjusted	1.00 (reference)	1.07 (0.86, 1.33)	1.16 (0.97, 1.39)
Fully adjusted*	1.00 (reference)	1.08 (0.87, 1.36)	1.19 (0.99, 1.43)

*Fully adjusted model includes age, gender, diabetes, systolic blood pressure, diastolic blood pressure, antihypertensive medications, body mass index and prevalent coronary heart disease, low density lipoprotein, high density lipoproteins, triglycerides, lipid lowering medication use, and smoking status.
